# Polyamines as a Universal Language of Host–Microbiota Symbiosis

**DOI:** 10.34133/research.1184

**Published:** 2026-03-06

**Authors:** Xiuyu Fang, Yan Guo, Jia Huang, Meimei Zhang

**Affiliations:** ^1^ Guangdong Provincial Key Laboratory of Animal Molecular Design and Precise Breeding, School of Animal Science and Technology, Foshan University, Foshan 528225, China.; ^2^ College of Wildlife and Protected Area, Northeast Forestry University, Harbin 150040, China.; ^3^ State Key Laboratory of Swine and Poultry Breeding Industry, Guangdong Laboratory of Lingnan Modern Agriculture, College of Animal Science, South China Agricultural University, Guangzhou 510642, China.

## Abstract

Polyamines are ancient metabolites that serve critical functions in maintaining epithelial integrity, regulating immune response, and supporting healthy aging. The gut microbiota actively synthesizes and converts polyamines, while host factors such as inflammation, barrier function, and nutritional status dynamically modulate this metabolic network. Disruption of this host–microbiota axis reduces polyamine availability, impairs barrier function, and exacerbates inflammation. In contrast, polyamines exert protective effects by promoting epithelial repair, modulating macrophage and T-cell responses, and enhancing autophagy-mediated tissue renewal and longevity. Recent advances in engineered probiotics, microbial small RNAs, and postbiotics further highlight the therapeutic potential of precisely modulating polyamine metabolism in clinical contexts such as inflammatory bowel disease, metabolic syndrome, and neurodegenerative conditions associated with aging.

Polyamines, among the most ancient and resilient small molecules, play a pivotal role in cellular proliferation, immune regulation, mitochondrial homeostasis, and aging processes [1]. One of the most exciting discoveries in recent years reveals that the gut microbiota acts not merely as a passive source of polyamines but also as an active regulator of their systemic homeostasis. This insight compels us to re-evaluate the metabolic symbiosis between host and microbiota and opens new avenues for understanding inflammatory diseases, cancer, metabolic disorders, and healthy aging. The gut microbiota contributes to polyamine pools through a complex network of biosynthetic, interconversion, and regulatory pathways [2]. Typical gut bacteria, including *Bacteroides*, *Lactobacillus*, and *Bifidobacterium*, harbor core enzymatic systems such as ornithine decarboxylase and agmatine deaminase [3]. These metabolic capacities enable the conversion of amino acid precursors into putrescine, spermidine, and spermine, thereby establishing community-level metabolic networks based on “cross-feeding”. In such metabolic networks, polyamine intermediates produced by one bacterial species can be assimilated and further modified by other members. For example, spermine generated by one taxon may be converted into putrescine by a second. This intermediate can then be elongated into spermidine or spermine by a third species. Consequently, the overall polyamine profile reflects the integrated metabolic capacity of the microbial community rather than the abundance of any single taxon. This distributed metabolic cooperation underlies the stability and functional resilience of the gut ecosystem. At the same time, it reveals critical vulnerabilities that can be disrupted by dietary changes, inflammation, antibiotic exposure, or other environmental disturbances. However, this intricate microbial metabolic network does not function independently. Instead, it is tightly coupled to host physiology through continuous, bidirectional interactions.

In this dialog, host physiology exerts profound feedback regulation over microbial polyamine metabolism. The host’s inflammatory state, intestinal barrier permeability, and nutrient availability collectively shape the microbiota’s capacity to synthesize and interconvert polyamines [4]. In disease states, host polyamine metabolism undergoes marked alterations [3]. For instance, during bloodstream infection, bacterial proliferation elevates levels of acetylated polyamines, while the disruption of the polyamine-producing enzyme SpeG reduces bacterial growth and attenuates disease progression [5]. Moreover, under pathological conditions such as colitis, the abundance of polyamine-producing bacteria is markedly diminished. This reduction leads to impaired polyamine metabolism, compromised intestinal barrier function, and exacerbated mucosal injury [6]. This dynamic establishes a classic vicious cycle of inflammation, polyamine depletion, barrier dysfunction, and microbial dysbiosis, forming a critical pathological basis for numerous chronic gastrointestinal disorders.

Polyamines exert broad and profound regulatory effects on host physiology. Intestinal epithelial cells are highly dependent on spermidine to maintain tight junction proteins, mitochondrial integrity, and mucus layer renewal [7]. Conversely, a polyamine-deficient intestinal epithelium rapidly exhibits barrier disruption [1]. Immune cells such as macrophages are also highly sensitive to polyamines. Putrescine and spermidine can reshape their metabolism, promoting a more reparative M2 phenotype and suppressing excessive inflammatory responses [8]. Polyamine metabolism in macrophages contributes to the maintenance of colonic epithelial homeostasis, with polyamines serving as a critical molecular bridge linking immunometabolism to epithelial repair [9,10]. Moreover, polyamine biosynthesis is essential for the initial activation of T cells [11]. More intriguingly, accumulating evidence links polyamines to lifespan extension, tissue regeneration, and antiaging, largely through their roles in promoting autophagy and maintaining mitochondrial quality control [12]. These pleiotropic effects depend both on local polyamine concentrations and their multi-tissue distribution, which are collectively shaped by the synthetic capacity of the gut microbiota, epithelial transport mechanisms, and the degradation kinetics of host enzymes [13].

Building on these advances, therapeutic strategies targeting polyamine metabolism are poised to offer marked clinical potential. Engineered probiotics may serve as “metabolic factories” capable of high-yield production of spermidine or agmatine, enabling more stable and controllable polyamine delivery. Professor Ren and his colleagues [14] uncovered a groundbreaking mechanism by which *Lactobacillus murinus*-derived small RNAs suppress host polyamine metabolism by inhibiting polyamine metabolic enzymes, thereby establishing a new paradigm of microbial RNA-mediated regulation of host polyamines in colonic diseases. Compared to traditional probiotics, these “functional microbes” hold promise as a new generation of precision nutrition tools [15]. Concurrently, the emergence of postbiotics has opened novel application scenarios for polyamines [16,17]. For instance, stable spermidine derivatives or gut-targeted microbial fermentation products offer enhanced safety and greater precision of delivery. Dietary interventions remain equally important. Diets enriched in arginine, fructooligosaccharides, resistant starch, or polyphenols can systematically enhance polyamine production by reshaping microbial metabolic pathways, thereby improving host health.

Despite these promising prospects, several fundamental scientific questions remain unanswered. Identifying which microbial communities predominantly govern the synthesis and turnover of the endogenous polyamine pool would lay the groundwork for microbiota-targeted interventions and precision nutritional strategies. Determining whether tissue-specific polyamine requirements exhibit circadian rhythmicity could have important implications for chrono-nutrition and time-dependent therapeutic modulation. At a broader level, elucidating how polyamine signaling integrates with short-chain fatty acids, bile acids, and tryptophan metabolism will be essential for building comprehensive metabolic regulatory networks from a systems biology perspective. In addition, polyamines exhibit clear double-edged properties in inflammatory and tumor microenvironments. While they are indispensable for macrophage-mediated resolution of intestinal inflammation, these same molecules can be co-opted within tumors to drive the polarization of tumor-associated macrophages, thereby facilitating tumor progression and immune escape [18,19]. Achieving spatiotemporally specific regulation of polyamine signaling therefore represents a critical hurdle for the development of safe and effective targeted therapies [20].

Overall, the gut microbiota–polyamine metabolism axis offers a new perspective on host–microbiota symbiosis. Moving beyond a simple metabolic supply–demand relationship, it operates as a sophisticated, multilevel communication system that spans barrier integrity, immunity, metabolism, and aging. Understanding and manipulating this system holds promise for delivering novel solutions to inflammatory diseases, metabolic syndrome, neurodegenerative disorders, and even extending healthy lifespan. Taken together, current findings converge on a unifying conclusion: rather than functioning merely as metabolites, polyamines constitute one of the most promising molecular “languages” mediating communication between the host and its microbiota (Fig. 1).

**Fig. 1.**
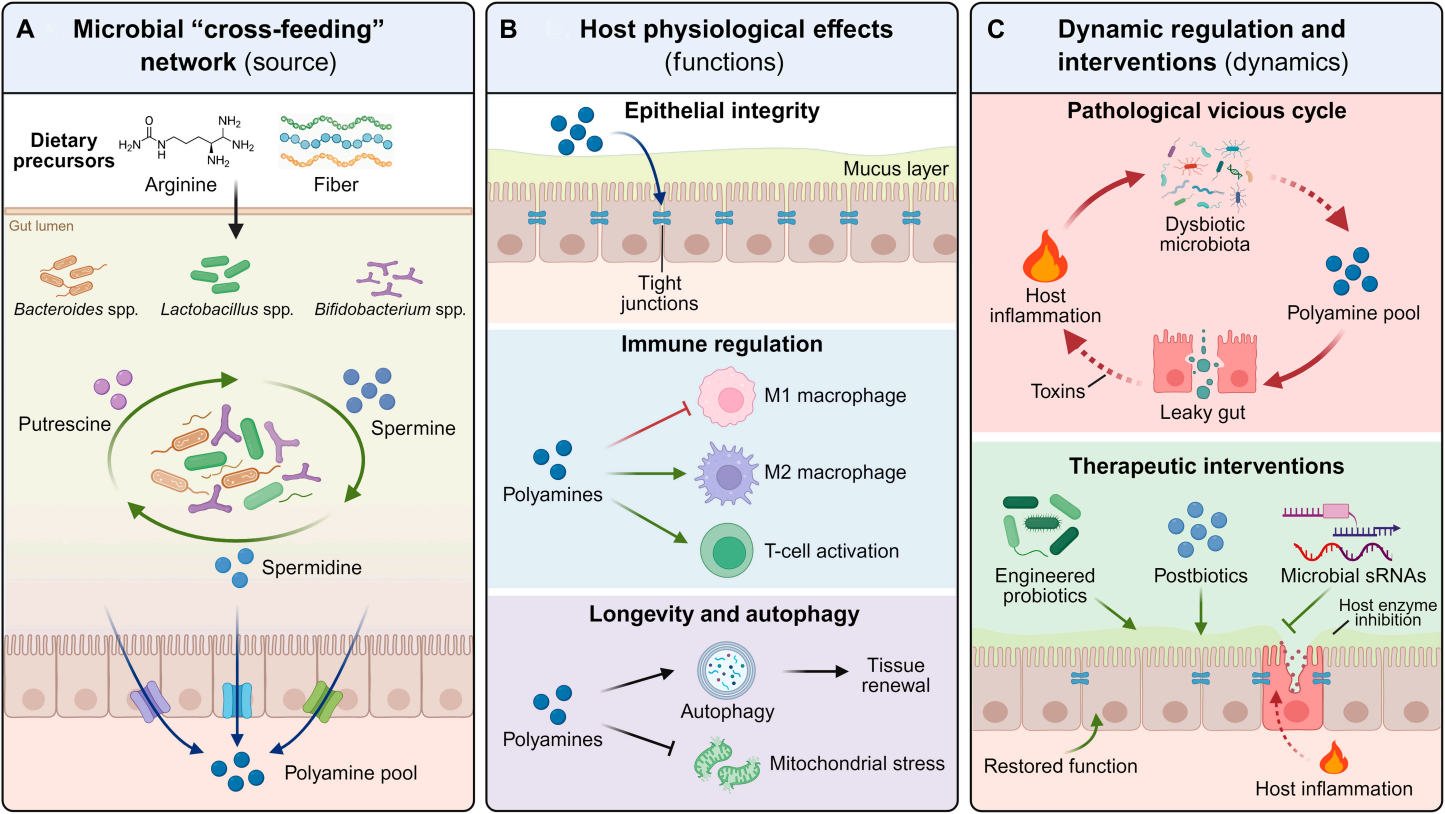
The gut microbiota–polyamine metabolic axis: cross talk, physiological functions, and therapeutic modulation. (A) Microbial biosynthesis and cross-feeding network. The gut microbiota functions as a metabolic factory in the intestinal lumen. Dietary precursors (e.g., arginine and fibers) serve as substrates for a diverse community of bacteria, including *Bacteroides*, *Lactobacillus*, and *Bifidobacterium*. Through a mechanism of metabolic “cross-feeding”, intermediate metabolites are exchanged and processed between different bacterial species, collectively generating a robust luminal pool of polyamines (putrescine, spermidine, and spermine). (B) Multidimensional physiological effects on the host. Upon transport across the epithelial barrier, polyamines exert pleiotropic effects on host tissues. In the intestinal epithelium, they maintain barrier integrity by reinforcing tight junctions, promoting mucus layer renewal, and ensuring mitochondrial quality control. In the immune system, polyamines modulate immunometabolism, driving macrophage polarization toward a reparative M2 phenotype and supporting T-cell activation. Furthermore, they promote cellular longevity and tissue renewal through the activation of autophagy. (C) Dynamic regulation and therapeutic interventions. The axis is subject to bidirectional regulation. Under pathological conditions, host inflammation inhibits microbial polyamine synthesis, leading to polyamine depletion, barrier disruption, and aggravated inflammation. Conversely, precision interventions such as engineered probiotics, postbiotics, and microbial small RNAs (sRNAs) offer novel strategies to restore the polyamine pool, repair the barrier, and resolve inflammation. Green solid arrows indicate activation or metabolic flow; red dashed arrows indicate inhibition or negative feedback.
